# On the Development of the Teeth, and on the Nature and Import of Nasmyth's "Persistent Capsule."

**Published:** 1854-04

**Authors:** Thomas H. Huxley


### ARTICLE XIV.

*On the Development of the Teeth, and on the Nature and Import of Nasmyth's "Persistent Capsule."* By THOMAS H. HUXLEY, F. R. S.

I AM desirous of setting forth in the course of the following pages, as concisely as may be, the principal results to which I have been lately led in the course of working over the development of the human and of some other teeth. I have directed my investigations, not to the general phenomena of dentition, our knowledge of the course of which, firmly established many years ago by Professor Goodsir, has not been affected, so far as I am aware, by any subsequent investigations, but to those points of structure and development upon which every writer, from the time of John Hunter to the present, seems to have formed, with more or less plausibility, an opinion of his own, different from that of all others.

I must suppose such a knowledge of the general course of development of the teeth as may be found in the ordinary hand-books of physiology—my limits allowing no unnecessary disquisition—and proceed at once to the questions whose discussion I am about to attempt. These are, firstly: What are the three structures which are concerned in the development of the teeth, viz. the pulp, the capsule, and the enamel organ, *morphologically*, or in relation to the parts of the mucous membrane from which they are developed?

Secondly: What is the relation of the dentine, the enamel, and the cement, to these organs?

Thirdly: What is the relation of the histological elements which enter into the composition of the soft parts, to the dentine, enamel, and cement, which are formed from or within them.

These questions, I think, involve all the essential points connected with the teeth. Having endeavored to answer them, I shall inquire with what other organs of the animal the teeth correspond.

1. ***The nature of the pulp, the capsule, and the enamel organ, with relation to the mucous membrane from which they are developed.***

The teeth are developed in two ways, which are, however, mere varieties of the same mode in the animal kingdom.* In the first, which may be typified by the mackerel and the frog, the pulp is never free, but from the first is included within the capsule, seeming to sink down as fast as it grows.

In the other the pulp projects freely at one period above the surface of the mucous membrane, becoming subsequently included within a capsule formed by the involution of the latter; a marked instance of this mode of development occurs in the human subject. The skate offers a sort of intermediate stage.

If the thick and opaque, colored, mucous membrane of the jaw of the mackerel be torn away, and the alveolar edge of the jaw be then examined with a low power, minute germs will be seen to be imbedded in the substance of the jaw, among the large, fully-formed teeth. It was an oval mass, about $\frac{1}{60}$th of an inch in long diameter; its upper part was roofed as it were by the epithelium of the gum; its sides were constituted by a continuation of the basement membrane of the mucous membrane of the mouth; within this was a homogeneous substance, containing numerous oval or rounded nuclei, about $\frac{1}{5000}$th of an inch in diameter, and continuous with the lowest layer of the epithelium of the mouth. In the centre appeared a large conical mass, nearly as long as the sac, the proper tooth pulp. Pointed above, it widened below, and then gradually contracted again, so as to form an almost hemispherical lower extremity, which was united to the base of the sac by a narrow neck. In the upper part of the papilla the proper dental tissues had already begun to make their appearance; but below, a delicate membrane formed its outer boundary, and this passed directly into the basement membrane of the sac.

*** For the purposes of the present examination, I have taken the skate, the mackerel, the frog, the calf, and man, as accessible specimens of each of the great divisions of animals possessing teeth.**

It is clear then, that in this case the papilla is wholly a process of the derm (or that which in a mucous membrane corresponds to it) outwards, while the sac is a process inwards of the same structure; and that the homogeneous substance, with its imbedded nuclei between the two, corresponds with the epidermis or epithelium.

In the frog the same relations essentially hold good; the young teeth are here developed in minute sacs, which lie at the bottom of the dental groove in the upper jaw. I could never detect any free-projecting pulps (nothing, therefore, corresponding to the papillary stage in the human tooth,) but the smallest and youngest rudiments of the teeth I found were oval or rounded sacs, $\frac{1}{180}$th of an inch long, containing an oval papilla, about one-fourth shorter. Externally, these were bounded by a strong structureless basement membrane, which enclosed a homogeneous substance containing nuclei in its cavities. These were rounded, and very close together, next to the basement membrane, but became transversely elongated in the inner layers and next to the pulp. This last was bounded by a structureless membrane, which at its narrow base became continuous with the basement membrane of the capsule.

In the frog, then, the relations of the pulp and of the capsule are the same as in the mackerel.

In the skate, as is well known,* the young teeth are developed in longitudinal rows within a deep fold of the mucous membrane of the mouth, behind the jaw. So far as my examinations go, however, I find that this is not a mere simple fold, such as it has been described to be; but its two walls behave just in the same manner as those of the primitive dental groove in man—that is, they become closely united in lines perpendicular to the direction of the jaw, so that partitions are formed between every two rows of teeth—transverse partitions again stretch between the separate teeth of each row, but these did

* See Blake's "Essay," &c. 1801, in which the essential peculiarities of the development of the teeth in the shark and skate, and their mode of advance, are very well pointed out. He refers to Herissant and Spallanzani as having anticipated him.

not appear to me to be complete, terminating by an arcuated border below. Each longitudinal canal, therefore, answers to a single elongated mammalian follicle, or to that prolongation of the alveolar groove from which the posterior permanent molars are formed in man (*see* Goodsir,) only the process does not go so far as in this case, the separate capsules remaining imperfect anteriorly and posteriorly. The lateral walls of the capsule, however, seem to me to have as much (or as little) "organic connection with the pulp and attachment to its base" as in man, and the process seems to correspond with something more than the "first and transitory papillary stage of the development of the mammalian teeth."*

Each pulp is invested by a very distinct basement membrane, whose continuity with that of the mucous membrane of the follicle is very obvious. The epithelium of the follicle forms a thick layer, which sometimes, when the upper wall is stripped back, adheres to it—sometimes remains as a cap investing the papilla. Even when the latter does not take place, shreds of the epithelium frequently adhere to the papilla in the form of irregular, more or less cylindrical nucleated cells; as often, however, the papilla, whether any of the proper tooth substances be formed or not, has nothing adherent to it, but presents a perfectly smooth sharp edge. Other portions of the epithelium, particularly towards the bottom of the follicles, are more or less altered and irregular, *frequently assuming the form of a stellate* tissue.

In the skate, then, the follicle is an involution of the derm, the papilla is a process of it, and the epithelium between the two becomes metamorphosed sometimes into a peculiar stellate tissue. The same essential relations prevail as before.

In man, some confusion has prevailed with regard to the homology of the various component parts of the tooth sac, though they might be readily enough deduced from the mode of development of the sac; however, it is, I think, not at all difficult to obtain perfect demonstration upon this subject.

* See Owen's "Odontography," p. 15.

If a young tooth capsule be opened (say of a fœtus at the seventh month,) whatever care may be exercised, it will always be found (Hunter, Bichât) that a space filled with a fluid exists between the inner surface of the capsule and the outer surface of the pulp—*the two are perfectly free from all adherence to one another*—the only substance between them, besides the fluid, being a more or less abundant whitish matter which sometimes adheres to the one and sometimes to the other (*see* Goodsir, *l. c.*)

If the tooth be very young, a structureless membrane, the m. preformativa of Raschkow (the basement membrane of Bowman,) may be traced over the whole surface of the pulp, or if calcific deposition have already commenced, it may be found readily enough at any rate in the lower unossified part; and it is not at all difficult to trace this in perfect continuity on the walls of the capsule—in fact into its basement membrane. The best way of seeing this is by detaching the whole sac from its alveolus, and then, laying it carefully open in a watch-glass, turn the capsule carefully back, transfer the whole to a glass plate, and cover it with a piece of thin glass. The continuity of the basement membrane of the pulp with that of the capsule is now evident enough under the microscope.

The wall of the capsule is often folded, and sometimes I have noticed villous processes, such as those described as vascular by Dr. Sharpey.* Not unfrequently the basement membrane of the capsule is quite naked, but I have sometimes observed a lining of short cylindrical nucleated epithelium cells upon it.

I have said that a whitish substance lies between the basement membrane of the pulp and that of the capsule. It is delicate and friable, but frequently forms a more resisting layer towards the pulp. On this surface I have found it to be composed of a layer of elongated, more or less cylindrical epithelium cells $\frac{1}{1000}$th of an inch in length, with or without nuclei, and adhering together in the direction of their short diameters. On the surface towards the capsule, on the other hand, this

* See also Goodsir, *l. c.* p. 17. In a child at birth "the interior of the sac had a villous, highly vascular appearance, like a portion of injected intestinal mucous membrane." See also p. 25 of the same admirable essay.

substance is composed of irregular cells united into a network, and very similar to those which have been described in the skate. The structure of this substance, and its relation to the basement membrane of the pulp, and of the capsule, clearly indicate that it is nothing more than the altered epithelium of these organs.* It is the so-called "*enamel organ*" of authors, and very wonderful figures and descriptions indeed have been given of it in various works upon the teeth. The only detailed,† and at the same time, as it seems to me, completely accurate account I have met with of this so-called enamel organ, is the very clear and admirable description by Mr. Nasmyth, contained in his posthumous work, "*Researches on the Development, Structure, and Diseases of the Teeth,*" 1849. The merits of this gentleman have met with such scant justice that I cannot do better than let them speak for themselves in this place; those who work over the subject hereafter will not fail, I think, to acknowledge them as I have done.

*Development of the Formative Organs of the Teeth, Follicular stage.*—"At an early period of the follicular stage when the apex of the papilla rises above the level of the surrounding fence of mucous membrane, a small quantity of whitish matter may be detected in the groove between the papilla and the follicle—this is the *enamel organ.* Not unfrequently the whitish matter has the appearance of granules which seem to have been separated from the surface of the follicle. These granular masses have

* Goodsir ("Edin. Med. and Phys. Journal," 1839) and Todd and Bowman ("Physiological Anatomy,") state very distinctly that the pulp is an ordinary papilla, and the capsule an involution of the mucous membrane, and the latter justly described the membrana preformativa of the pulp as a basement membrane (p. 175,) but they consider the "stellate tissue" and the enamel organ to be the "wall of the sac itself." Kölliker ("Mikr. Anat.," p. 101) expresses the same opinion.

† Mr. Tomes ("Lectures," &c., 1848) appears to me to have described the enamel organ very accurately, but he has, I think, failed to distinguish the proper enamel organ or epithelium of the sac from the submucous cellular tissue—the latter is his "reticular stage of the enamel pulp," the former his "second stage" or "stellate tissue," while what he calls the "transition part," p. 99, is, I think, the dense superficial layer of the capsule, very well described by Mr. Nasmyth (*vide infra*) as "the internal lamina of the dental capsule."

Professor Kölliker ("Mikr. Anat.," p. 99 B) appears to me to have fallen into the same error.

a pearl white aspect, and are soft and friable. Under the microscope they are seen to be composed of cells which separate from one another upon the slightest compression. The cells offer considerable variety in respect of size and shape, some being small and round, others large and flattened, and furnished at one extremity with a delicate prolongation; while others again are elongated and narrow, and have a defined and regular margin. They contain nuclei and nucleoli, and are covered on their interior by minute granules, which are also found in considerable abundance in their interstices."—p. 104.

"In the numerous examinations which I have made of the stages of growth of the teeth here described, the enamel organs did not appear to me to be attached either to the papilla or to the surface of the follicle. This may probably arise from the circumstance that all the embryos which I dissected had been kept for some time in diluted spirits of wine."—p. 105.

He then quotes Raschkow's account of the structure in the lamb and calf, and goes on to say,—

"In my own investigations made with the aid of one of the best microscopes of modern construction, and with a magnifying power of one-tenth of an inch focal distance, I found the enamel substance to be composed of cells of three different kinds.

"The first kind of cells are found in the interior of the organ, and compose its loose, soft, and easily compressible texture. They are flattened and triangular in form, and connected to adjacent cells by means of delicate filaments prolonged from one of their angles. These appendages have no analogy with the filaments of areolo-fibrous tissue, as declared by Raschkow. I have seen them in connection with the cells of other tissues, and the error on the part of this observer must have arisen from the use of low microscopic powers.

"The second kind of cells are oval in shape, and form an envelope to the preceding: they are situated both upon the superficial and deep aspect of the latter.

"The third kind of cells occupy the deep stratum of the enamel organ, lying in contact with the dental papilla. They are narrow and oblong in shape, and are arranged closely side by side; one of their extremities being in relation with the papilla, the other being directed outwards. They are firmly connected together, and have a radiated position in respect of the papilla. It is to the layer formed by these cells that Raschkow has assigned the name of enamel membrane. Taking this view of the construction of the enamel organ, I cannot perceive any grounds for the division of it into two parts suggested by the description of Raschkow. It is obviously nothing more than a single organ, and the difference in the form and arrangement of the cells must simply be regarded as a transition of the first and second kinds into those of the third—the latter being in the state of preparation for the reception of the calcareous salts.

"The mucous membrane which rises in the form of a ring fence around the papilla developed from the dental groove is the future *dental capsule.* At an early period it is difficult to determine to what extent the internal surface of the growing follicle differs from mucous membrane. That it does so may be inferred from the change in function which it assumes; and at a later period, when the follicle is about to close, the difference in its organic character becomes strikingly obvious. For example, it is white, silvery, loose, and rugous, and easily falls into folds, and, under the microscope, offers the appearance of a number of minute cells possessing characters widely different from those of the epithelium.

"A portion of the internal lamina of the dental capsule, placed under the microscope, shows it to be composed of layers of cells loosely arranged, and separated by interspaces equal to half the diameter of the cell. The cells are oval in shape, and provided with one or more distinct nuclei, and they contain in their interior a small quantity of granular matter. The internal lamina of the dental capsule maintains but a slight degree of adhesion with the enamel organ, and possesses no vessels. Subjacent to it is a network of blood-vessels, supported by a web of areolo-fibrous tissue formed by the interlacement of fine homogeneous filaments, among which nucleated cells are not unfrequently observed."—p. 107.

*Saccular Stages.*—When the sac closes—

"The space between the pulp and the sac becomes filled with a fluid secretion which distends its cavity, and often produces a conspicuous enlargement in the situation of the tooth."—p. 108.

"On the part of the capsule corresponding with the sides and neck of the crown is a flat portion of the enamel organ, which is destined to the formation of the enamel in that situation. This lamina has a well defined inferior border at a later period in the growth of the enamel organ; the appearance which it presented of a gelatinous mass is lost, and the substance contracts into a membranous layer. At this time also the prominences from the internal surface of the capsule have enlarged, and have become vascular and more closely adherent to the enamel organ. Some writers have inferred from this appearance that the enamel organ itself becomes vascular,* but this is not the fact; it is simply that portion of the capsule which lies in contact with the enamel organ that presents the vascularity referred to.

"The dental capsule being originally, as we have seen, a production of the mucous membrane of the alveolar groove, is attached by its external surface to the neighboring soft parts by means of loose areolo-fibrous tissue. Blood-vessels ramify very freely in this tunic, and from the interlacement which they then form, numerous capillary loops are given off, which extend into the superficial portion of the membrane. These vascular loops are separated from the enamel organ by a delicate layer of cells, the characters of which have been already explained.

"Not the least interesting of the features attendant upon the development of the teeth is the relation which the capsule bears to the pulp and to the tooth at various periods of its growth. In the follicular and early periods of the sacular stage, previously to the commencement of the formation of the ivory, the capsule is continuous with the base of the dental papilla;† and at a subsequent period, when the ivory of the crown forms a complete covering to the pulp, the same arrangement takes place. But at a more advanced stage in the growth of the tooth, when its formation has proceeded beyond the limit of the crown, the capsule attaches itself closely around the neck, and the connexion of the two structures is so firm, that every attempt to effect their separation generally results in the laceration of the membrane. The continued growth of the tooth carries the capsule upwards with the rising alveolus to the under part of the gum, which now stretches over it; when pressed upon by the surface of the crown, it becomes atrophied and absorbed. No portion of the capsule seems to pass down into the alveolus."—p. 110.

Everything that I have seen confirms this admirable description as to matters of fact, and the only objections I shall have to offer are to certain of Mr. Nasmyth's conclusions.

In man, then, as in the skate, the mackerel, and the frog, the tooth-pulp is a dermic process bounded by its basement mem-

*Raschkow, in a note appended to his Researches, remarks that he has observed the enamel organ to receive blood-vessels in certain parts, and believes the parenchyma of the organ to be pervaded by capillary vessels. The conclusion which he deduces from this observation is, that the enamel organ was from the beginning joined to the capsule.

†It passes upwards over it, forming a distinct envelop, separated from the layer of mucous membrane externally.

brane; the capsule is an involution of the derm, bounded by its basement membrane; and the epithelium of these organs lies between them, having in this case received the name of "*enamel-organ*," from the supposition that the enamel was developed by the calcification of its elements. Of this, however, I shall speak below.

There is an important difference between the dental sac of the calf and that of man, which has given rise to much confusion.

The "actinenchymatous" tissue (Raschkow) of the former does not at all correspond with the stellate tissue of the latter, as has been assumed by all writers. In fact, in the calf the wall of the capsule is separated by only a very narrow space from the surface of the pulp, and this space is completely filled up by elongated cylindrical epithelium cells, which glue the capsule to the pulp. Between the basement membrane of the capsule and the alveolar wall, indeed, there is a very wide interval (see Owen, *l. c.*, pl. cxxii, *a.* fig. 9 *e*) occupied by Raschkow's actinenchyma. This, however, is nothing more than the loose submucous cellular tissue of the gum, similar to that so well described by Mr. Nasmyth in the wall of the capsule of man. Professor Owen says (*l. c.*, Introduction, p. lix,) that "no capillaries pass from the capsule into the actinenchymatous pulp of the enamel." But those which I have examined do not bear out this statement; in fact, this tissue presents one of the most beautiful and obvious vascular networks with which I am acquainted.*

The true homologue of the "enamel organ" in man therefore, in the calf, is not the actinenchymatous tissue, but the thin layer of epithelium between this and the pulp. The general relations of the different dental organs are, in other respects, the same in the calf as in man.

I may now proceed to the second question. *What is the relation of the proper dental tissues to the three organs of the tooth capsule?*

The answer is shortly this. Neither the capsule nor the

* Blake, who wrote in 1801, mentions the vascularity of the "spongy" outer membrane of the tooth sac in the calf; he says it is "very vascular."—p. 81.

"enamel-organ" take any direct share in the development of the dental tissues, all three of which—viz. enamel, dentine and cement—are formed beneath the membrana preformativa, or basement membrane of the pulp. In proof of this assertion, I have to offer the following facts:—If in a human fœtus of the seventh month, a dental capsule (say of an incisor) be treated as I have above described, it will generally happen that the surface of the young tooth-cap appears quite smooth under a low power; or it may be that a few of the elongated cells of the "*organon adamantinæ*" adheres to it. In any case the adhesion is loose, and these cells may be readily detached. Under a higher power the surface of the upper part of the ossified cap appears reticulated, the meshes being about 1-5000th of an inch in diameter. At the lower part, where only a thin layer of dentine is formed, this appearance is less distinct, but the surface is somewhat wrinkled, the wrinkles sometimes forming large and pretty regular meshes. Viewed in profile, these wrinkles are seen to be produced by the folding of a delicate structureless membrane, which is continuous below with the *membrana preformativa.* Towards the apex the tooth substance is almost too opaque to make much out of it; the yellowish enamel, however, can generally be distinguished from the dentine.

Now, while the object is under a low power of the microscope, add some strong acetic acid; a voluminous transparent membrane will immediately be raised up in large folds from the whole surface of the tooth. If the acetic acid be pretty strong, it soon softens the substance of the tooth a little, and then a slight pressure exhibits very distinctly the *ends of the enamel fibres under this membrane.* There can be no question about this fact, as I have been able to demonstrate it to the satisfaction of my friends, Mr. Busk and Professor Quekett. The membrane is about 1-2500th to 1-1600th of an inch thick, perfectly clear and transparent, and under a high power exhibits innumerable little ridges upon its outer surface, which bound spaces sometimes oval and sometimes quadrangular, and about 1-5000th of an inch in diameter. Furthermore, at its lower edge this membrane gradually loses all structure, and passes into the *membrana preformativa.** *In fact it is the altered membrana preformativa itself*, no trace of which has ever yet been found in the locality in which, according to the prevalent hypothesis upon the development of the teeth, it should exist—viz. between the enamel and the dentine.

In the calf † a similar membrane may be denominated, but it is much more delicate, and I have not seen the peculiar areolæ upon its surface.

In the frog, in which the layer of enamel is very thin and structureless, the membrane may be very readily demonstrated by the action of dilute hydrochloric acid, which in this animal, as in the mackerel and skate, dissolves out the enamel layer at once, while it only acts gradually upon the dentine.

In all these animals I have examined the smallest teeth I could find perfectly entire, without any rough mechanical treatment, which I should think would destroy the delicate membrane.

In the frog, its surface is in parts *reticulated as in man ;* in the mackerel and skate I have been unable to find any such reticulation. In both these the enamel forms a conical cap of almost structureless or obscurely fibrous substance at the extremity of the tooth, while the layer upon the body of the tooth is very thin.‡ In the skate it is thick, dense, yellowish, structureless, and perfectly smooth; but in the mackerel it is developed upon the lateral edges of the young tooth into sharp notched processes; lines stretched across the body of the tooth from these, not only unlike the contour lines one sees on the enamel of a young human tooth.

A membrane, corresponding with that which has been de-

* It is stated, by all the writers on the subject whom I have consulted, that the membrana preformativa is the first portion of the tooth which ossifies. This statement, however, is never supported by evidence ; and my own observations lead to precisely the reverse conclusions.

† *See* Hassall, Micr. Anatomy, p. 318.

‡ As this "dense exterior layer" may be dissolved out by dilute acid, leaving the "membrana propria of the pulp," which is very much thinner, standing, it is quite clear that it is not "formed by the calcification of the membrana propria of the pulp, which therefore precedes the formation of ordinary dentine." (*Odontography*, p. 17.) Why should it not be called enamel? It has at least as much claim to this title as that of the frog.

scribed in the human subject then, is also found in members of each of the other groups of vertebrata which possess teeth. In the human subject, and in mammals, this membrane was discovered, and very accurately figured and described, fourteen years ago (that is, in January, 1839, in the Medico-Chirurgical Transactions,) by Mr. Nasmyth, under the name of the "persistent capsular investment." No question has ever been raised as to the right of Mr. Nasmyth to this discovery; but it is remarkable, that neither in Professor Owen's Odontography, which is the first subsequent work upon the teeth, nor in Professor Kölliker's Mikroskopische Anatomie, which is the last, is there any notice of Mr. Nasmyth's discovery. Kölliker, indeed (*l. c.*, pp. 76, 77,) describes the structure as "schmelzoberhäutchen," but his description is not so good as that of Nasmyth, and he states that it does not extend over the cement—Nasmyth having shown that it does. Unfortunately, however, the latter, like all who have succeeded him, misled by the supposed mode of development of the enamel from the enamel-organ, imagined that, as the "persistent capsule" was outside the enamel it could be nothing else than the membrane of the dental capsule; and hence the erroneous description of the adherence of the latter to the crown of the tooth, which I have already quoted. Had he chanced to examine a tooth before its eruption, he would at once have seen the incorrectness of his hypothesis.

Since then this "Nasmyth's membrane" is identical, on the one hand, with the persistent capsule which lies external to both enamel and cement, and, upon the other hand, with the preformative membrane of Raschkow or otherwise with the basement membrane of the pulp; it is clear that all the tissues of the tooth are formed *beneath the basement membrane of the pulp;* in other words, they are all true dermic structures—none epidermic.*

*That the enamel is not formed directly from the enamel pulp might have been concluded from Professor Goodsir's observations (*l. c.*, p. 25.) He says, "The *absorption* (in the granular matter) goes on increasing as the tooth substance is deposited, and when the latter reaches the base of the pulp, the former disappears, and the interior of the dental sac assumes the villous vas-

The third problem was, the relation of the histological elements of the soft parts (that is, as we now see, of the pulp) to the dentine, enamel, and cement.

Three theories have been prevalent as to the mode of development of the dentine. The first, the old *excretion theory,* need not be considered here, as it has been given up on all sides. The second, the Conversion theory, consists essentially in the supposition that the dentine is the "ossified pulp;" that the histological elements of the pulp become calcified and converted directly into the dentine—the arrangement of the elements of the dentine depending upon that of the elements of the pulp. This is the doctrine maintained by Blake, Schwann, Nasmyth, Owen, Tomes, Henle, Todd and Bowman, and, more or less doubtfully, by Kölliker and Hildebrandt.* The third theory is that contained in the remarkable phrase of Raschkow.

"Postquam . . . fibrarum dentalium stratum depositum est (quoted by Schwann) idem processus continuo ab externa regione internam versus progreditur *germinis dentalis parenchymate materiam suppeditante* . . . . Conversæ fibrarum dentalium flexuræ quæ juxta latitudinis dimensionem crescunt, dum ab externa regione internam versus procedunt sibi invicem appositæ continuos canaliculos effingunt, qui ad substantiæ dentalis peripheriam exorsi multis parvis anfractibus ad pulpam dentalem cavumque ipsius tendunt, ibique aperti finiuntur novis ibi quamdiu substantiæ dentalis formatio durat fibris dentalibus aggregandis inservientes."

The dentinal substance, that is, is deposited within the pulp beneath the membrana preformativa in definite masses (Raschkow calls them fibres, to which, indeed, under a low power they have a remarkable resemblance,) the gaps between which eventually constitute the dentinal tubules. This, if a name be wanted, might be called the Deposition Theory, and is especially charcular appearance of a mucous membrane. This change is nearly completed about the seventh or eighth month." It will not be said, however, that the growth of the enamel ceases at the seventh or eighth month.

* Dr. Sharpey, on the other hand, with characteristic caution, after citing the statements of some of the advocates of the Conversion Theory, adds, "We must confess that, after a careful examination of the human teeth, we have been unable to discover any of the above mentioned changes, except the enlargement of the more superficial cells of the pulp, and their elongations in the immediate vicinity of the dentine."—Quain and Sharpey, p. 988.

acterized by its asserting which the histological elements of the pulp do not enter *as such* into the dentine. The following description of the young dentine in the human subject holds good for all the animals that I have examined; and if it be true, I think the incorrectness of the Conversion Theory necessarily follows.

To justify my own method of procedure, however, I am necessitated to remark that I have been unable to verify the statement of Professor Owen (*l. c.*, Introduction, p. xxxix.,) that the teeth of man "will not yield a view of the cap of new-formed ivory and the subjacent pulp in undisturbed connection by transmitted light with the requisite magnifying power." On the contrary, I have found it sufficiently easy, by cutting off the half-ossified cusp of a young molar, or even by submitting an entire canine or incisor to slight pressure, to obtain a most distinct view of the pulp in undisturbed connection with the dentine, and in a profile view. Indeed, had other observers adopted this method, I do not think they would have been led to consider the lacunæ in young dentine, whose true nature was demonstrated by Raschkow, as metamorphosed nuclei of the pulp.

When the ossifying boundary of a tooth-pulp is examined in the way which I have here pointed out, it is seen that where dentification has not begun, the membrana preformativa is in immediate contact with the substance of the pulp, composed of a homogeneous transparent base, in which closely arranged "nuclei" are embedded. These are rounded or polygonal, apparently vascular; contain one or more granules, and are about $\frac{1}{2500}$th—$\frac{1}{3500}$th of an inch in diameter. Passing towards the ossifying edge, we see in the profile view a clear, more strongly refracting layer, gradually increasing in thickness, which begins to separate the proper substance of the pulp from the membrana preformativa. This is at first quite structureless to all appearance, both in this view and in one perpendicular to its surface. When it has attained a thickness of $\frac{1}{2500}$th of an inch, however, it acquires a sort of mottled appearance in the profile view, while superficially numerous very minute irregular cavities, about $\frac{1}{5000}$th of an inch apart present themselves. In a thick portion of the dentine ($\frac{3}{5000}$ths) these cavities are very readily seen in the profile view to be elongated into canals; superficially they are rather larger; and as they run somewhat obliquely, it may very readily happen that, unless the focusing of the microscope be very careful, one will run into the other, and so produce the appearance of fibres described by Raschkow.

This young dentine is as transparent as glass. No trace of "nuclei" can at any time be discovered in it; the bodies which have been described as such being, as I have said, simply lacunæ; nor, if strong acids be used so as to dissolve out the calcareous matter, are any nuclei brought to light, though those which exist in the pulp became much more distinct, and even coarse, in their outlines. Again, if to a pulp thus treated, a weak solution of iodine be added, the nitrogenous substance of the pulp is immediately colored deep yellow, the nuclei themselves becoming brown; but the dentine remains pale, except that here and there a yellow process of the matrix of the pulp may be seen stretching a little way into one of the canals of the dentine. I have only observed this, however, once. I believe that these facts afford sufficient demonstration that the pulp is *not* converted directly into the dentine, and that, the structure of the latter does not depend upon the calcification of pre-existing elements.

I am the more satisfied with this negative evidence, as in young bone it is easy to demonstrate the "nuclei" in the lacunæ by the aid of acids, &c.

As to whether the perpendicularly crowded "nuclei" of the pulp under the dentine disappear, or whether they are merely pressed inwards, I cannot pretend to offer a decisive opinion. The former supposition, however, if we may judge by the analogy of bone, appears more probable. Dentine, in fact, might be considered as a kind of bone, in which the lacunæ are not formed in consequence of the early disappearance of the nuclei, whose persistence for a longer or shorter period appears to be the sole cause of their existence in bone.*

*I have here no space to enter into the discussion of the various hypotheses and assertions, respecting the development of the dentine, made by the various authors, whose names I have cited. I trust it will not on that account be supposed that I have neglected to make myself acquainted with them. But there are two statements to which I must refer in confirmation of my own view.—

Still less can the enamel be produced by any *conversion* of a cellular structure. Between it and anything which can be called a nucleated cell it has on the outer side Nasmyth's membrane; on the inner, the layer of dentine, which in man is formed before it. The fibres of which it is composed are structureless, and almost horny; and I think we must be content for the present to consider its existence and its structure as ultimate facts, not explicable by the Cell Theory. It is particularly worthy of notice that in the skate the dermal teeth or plates on the upper surface of the head have as distinct a layer of enamel as those of the mouth, though in this case there is most assuredly neither rudimentary capsule nor "enamel organ."

In a morphological point of view, the relations of the cement show it to be homologous with the enamel. In a very beautiful section of a human tooth from Mr. Busk's cabinet, the upper portion of the cement exhibits in places a very distinct transverse striation, resembling its perfect enamel. But the transition of the one structure into the other is best exhibited in the young calf by the cement of the fang of a molar which had not cut the gum. Here it is a white substance, from which generally a fitting section can be cut only with some difficulty, in consequence of its friability. The layer is about 1-40th of an inch thick, and consists of an external delicate structureless Nasmyth's membrane; internal to which three-fourths of the thickness of the layer are formed by parallel fibres 1-5000th of an inch in diameter, quite structureless, and completely resembling enamel fibres, but absolutely enormous (as much as 1-60th of an inch) in length. These fibres were softened and rendered pale by the action of caustic ammonia. The inner fourth of the layer of cement was composed of an inextricably interlaced body of such fibres, united into a mass, which in some places was almost homogeneous, by calcareous salts, and containing here and there lacunæ 1-1600th of an inch in length, similar to those of bone. That this structure was the young cement is

The one is that by Dr. Sharpey already quoted; the other is the very just declaration (in italics) by Professor Kölliker (Handbuch, p. 386,) that "*the most careful investigation exhibits no trace of any elongation of nuclei*" in the peripheral cells of the pulp.

certain, inasmuch as no enamel is formed on the fang of the tooth, to say nothing of the presence of the lacunæ. On the root of the fang of the molar in front of this, which had cut the gum some time, and had come into use, the cement had the ordinary structure. It may be worth while to add that in these teeth the capsule, though closely connected with the outer surface of the fang, could be readily stripped from it, and then exhibited a layer of epithelium upon its inner surface, showing clearly that the cement was not derived from its ossification.

It may be concluded, then,—

1. The teeth are true dermic structures, formed by the deposit of calcareous matter beneath the basement membrane of a dermic papilla, or that which corresponds with one.

2. Neither the capsule nor the "enamel organ," which consists of the epithelium of both the papilla and the capsule, contribute *directly* in any way to the development of the dental tissues, though they may *indirectly.*

3. The histological elements of the pulp take no direct part (except, perhaps, eventually in the cement) in the development of the dental tissues, becoming either absorbed or being pressed in by the gradual increase of the latter. The Conversion Theory, is, therefore, as incorrect as the Excretion Theory, and the dentine is formed, not by ossification of the histological elements of the pulp, but by deposition in it, "parenchymate materiam suppeditante."

I have already exceeded my limits, and I must, therefore, dismiss my last point very concisely. The true homologues of the teeth in man are, I think, the hairs. As Hildebrandt says, "As the hairs in their bulb (sac,) so the teeth are developed in their capsules." The stage of the free papilla, which does not occur in the hairs of man, is absent in the teeth of the mackerel and frog, and, indeed, it would seem in the permanent dental capsules of man also.

Substitute corneous matter for calcareous, and the tooth would be a hair. The corticle substance of the hair contains canals not unlike those of the dentine; its relation to a dermal papilla is the same as that of the dentine;* for although it is univer-

* See Todd and Bowman, p. 175.

sally stated to be such, I think it can be shown that the hair shaft is *not* an epidermic structure but a dermic one.

Again, the so-called cuticle of the hair corresponds in all respects, except absolute and relative size, with the enamel—its inner layer with the enamel proper—its outer with Nasmyth's membrane. On the root of the hair the cuticle is not continuous with the proper epidermic cells, but with a structureless membrane, which occupies more or less distinctly the place of a *membrana preformativa.* The two root sheaths, again—true epidermic structures, but which do not enter all into the construction of the hair proper—represent the altered and unaltered portions of the "*enamel organ.*"

*Hairs* and *teeth*, then, are organs in all respects homologous, and true dermal organs. Under the same category, probably, will come feathers and the scales of fishes.

The nails, on the other hand, seem to be purely epidermic, at least according to Kölliker's account of their development (*l. c.*, p. 119;) and in that case they are the homologues of the root sheaths and enamel organs of hairs and teeth.

